# Automatic scent creation by cheminformatics method

**DOI:** 10.1038/s41598-024-82654-7

**Published:** 2024-12-28

**Authors:** Manuel Aleixandre, Dani Prasetyawan, Takamichi Nakamoto

**Affiliations:** Laboratory for Future Interdisciplinary Research of Science and Technology (FIRST), Institute of Integrated Research (IIR), Institute of Science Tokyo, 4259 Nagatsuta-cho, Midori, Yokohama, 226-8503 Kanagawa Japan

**Keywords:** Digital olfaction, Odor reproduction, Deep neural network, Mass spectrometry, Odor prediction, Cheminformatics, Machine learning, Cheminformatics, Mass spectrometry, Olfactory system

## Abstract

The sense of smell is fundamental for various aspects of human existence including the flavor perception, environmental awareness, and emotional impact. However, unlike other senses, it has not been digitized. Its digitalization faces challenges such as the lack of reliable odor sensing technology or the precise scent delivery through olfactory displays. Its subjective nature and context dependence add complexity to the process. Moreover, the method of converting odors to digital information remains unclear. This work focuses on one of the most challenging aspects of digital olfaction: automatic scent creation. We propose a method that automatically creates a desired odor profile with the addition of one specific odor descriptor. It is based on a deep neural network that predicts odor descriptors from the multidimensional sensing data, such as mass spectra and an odor reproduction technique using odor components. The results demonstrate that the proposed method can successfully create a scent with the desired odor profile and that its performance depends on the accuracy of the underlying odor predicting method. This opens up the possibility of automatic scent creation, allowing for the presentation of scents with specific odor profiles with an olfactory display.

**Supplementary Information:**

The online version contains supplementary material available at 10.1038/s41598-024-82654-7.

## Introduction

Although visual and auditory senses can be digitalized, olfaction cannot be integrated into cyber-physical space^1^. One key step in its digitalization would be the algorithmic design of scents with specific odor descriptors. Although perfumers can design sophisticated scents by blending ingredients, it requires long time and significant effort to obtain the intended scent. Moreover, acquiring the skill of scent creation takes a long period of time and involves many trials and errors^2^. Although previous works have been done in this area, they were focused on designing new odorous molecules with desired odor descriptors^3,4^ and most of the times they were not experimentally tested^5^. So far, no algorithm has been able to automatically create a desired scent.

However, recent remarkable progress on digital olfaction technologies^6–8^ can contribute to automatic scent creation. We already have two fundamental cheminformatics technologies to achieve this. The first one is the odor reproduction technique using odor components^9,10^. With this technique multidimensional sensing data (e.g. mass spectra) of scents are analyzed and decomposed into those of odor components and then we could reproduce the scents by blending those odor components. While our earlier research used odor components for reconstructing the mass spectra of existing scents^10^, here we take a fundamentally different approach by using them to design new scents. The second one is to predict the odor profiles (either odor descriptors or odor ratings). Although molecular descriptor parameters are often used to predict the odor profiles^11–15^, they cannot be normally applied to chemical compound mixtures and it requires prior knowledge of the substances in the mixture.

We have developed a method to predict the odor profiles from multidimensional sensing data (mass spectra)^16–18^. This method is appropriate since it is applicable to the mixture with high accuracy. Although we have already demonstrated that the mass spectrum corresponding to the odor profile can be obtained, the result is only numerical computation and the actual smell has not been generated^19^. Moreover, the present study differs from that earlier work in key aspects. We focus on essential oils, which introduce a different data nature compared to single molecular components commonly used in prior research^19^. Our sensory data are based on binary odor descriptors rather than continuous odor ratings^19^, that are more difficult to standardize across different domains (e.g., essential oils versus perfumes). Binary odor representations, on the other hand, are more natural and closely resemble human language, enhancing their practical utility. Additionally, we introduce odor components as an intermediary step in a generative process where as before they were used to reproduce existing odors^9,10^. In this paper, we created the actual scent with intended odor descriptors using odor reproduction and odor profile prediction techniques, followed by sensory evaluation^20^.

## System description

**Fig. 1:**
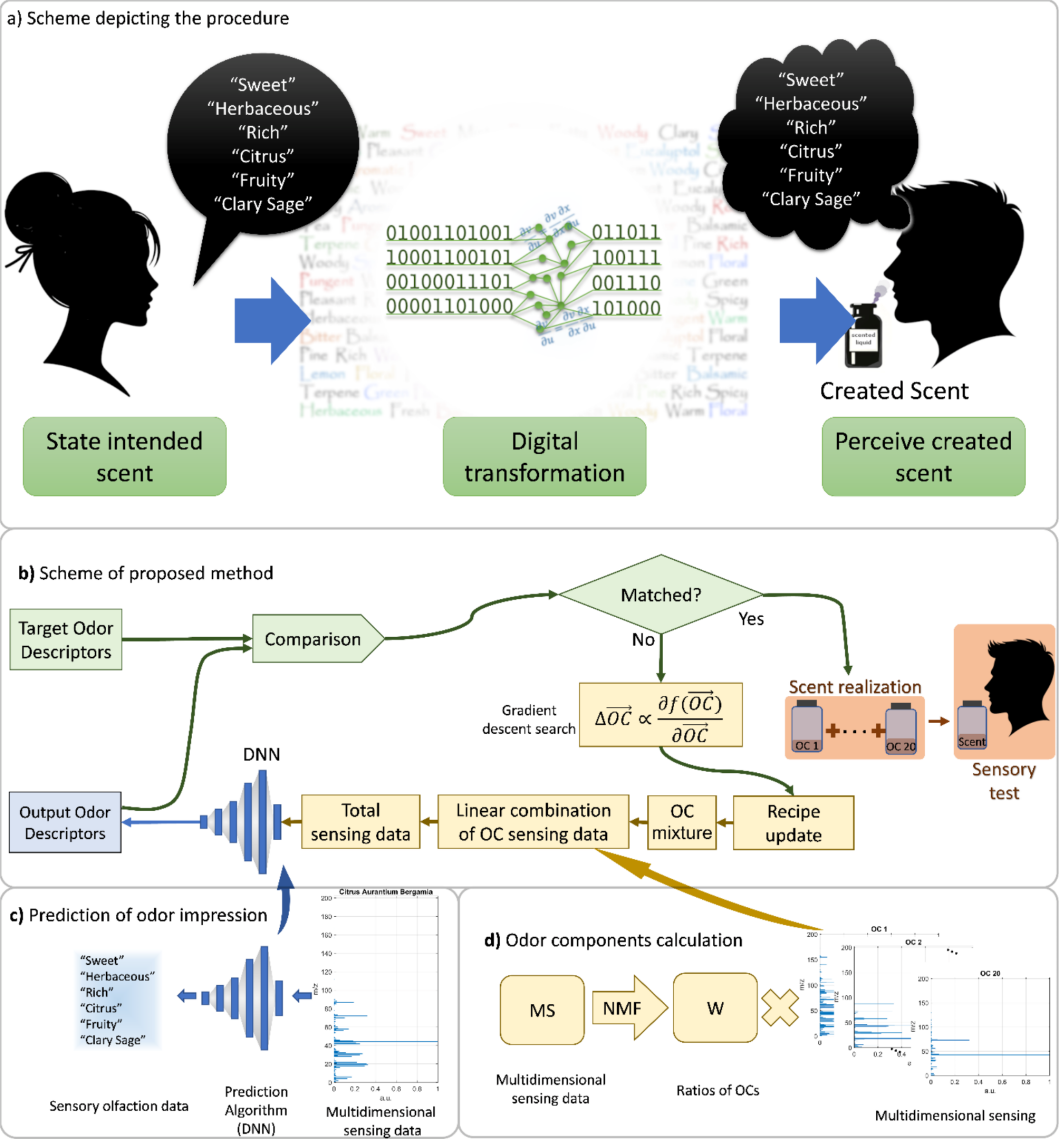
Scheme depicting the procedure. In panel (**a**) the general scheme of the algorithm is shown. In panel (**b**) the algorithm’s different parts are detailed where $\:\overrightarrow{OC}$ represents the combination of odor components that make up the created scent and $\:f\left(\overrightarrow{OC}\right)$ is the cost function to show the degree of matching. In panel (**c**) is the odor profile prediction algorithm and in panel (**d**) the odor component calculation.

Figure 1.a shows the system overview. First, a chosen odor profile is described and is input into the system. Then the algorithm designs a scented liquid that matches the described odor profile. This liquid is created and subsequently tested by human subjects.

We have three main parts to create scents, as depicted in Fig. 1: an Odor Component (OC) decomposition, an Odor Descriptors (OD) prediction algorithm, and a gradient descent search that integrates the two previous parts. Figure 1.b shows the system overview. First, a chosen set of odor descriptors is input into the system (target odor descriptors), using the three parts an odor component mixture is obtained and its predicted odor descriptors are calculated. The obtained odor descriptors and target odor descriptors are compared, and if they are equal, the odor component mixture is blended for human testing. Otherwise, the difference between the odor descriptors (target and obtained) is used to update the multidimensional sensing data (by modifying the odor component mixture) with a gradient descent search that propagates through the DNN and the odor component decomposition. This process is repeated until the convergence is achieved.

Figure 1.c shows the method to obtain the odor profile from a multidimensional sensing data, which, in our case, is mass spectrum data. It is mapped into a set of odor descriptors through a Deep Neural Network (DNN)^21,22^. Figure 1.d shows how the odor components are pre-determined by Nonnegative Matrix Factorization (NNF) method^23,24^. Thanks to the odor components we can reproduce the scent using a small set of odor components, although it could also be reproduced using all sampled essential oils. The scent can be actually reproduced using a multi-component olfactory display where each channel has an odor component^25^. The reproduction by the olfactory display is a next step of the current study.

Several types of sensing data can be used for the multidimensional sensing, such as, e-nose^26^, olfactory bulb^27^, sensory data^28^, and mass spectrum^29^. We selected the mass spectrum in this study since it provides a large amount of information and good reproducibility. Additionally, it is possible to accumulate many sample’s data easily because a mass spectrometer when used without gas chromatography, operates quickly. From an implementation standpoint, the property of linear superposition is essential. When dealing with mixtures, the number of possible combinations is too large to handle practically, making it nearly impossible to obtain data for all combinations. The linear superposition property enables us to use minimum data without many actual measurements. The mass spectrum of a mixture can be simply calculated as the linear combinations of the mass spectra of all ingredients when the mixture composition is known.

### Data

**Fig. 2:**
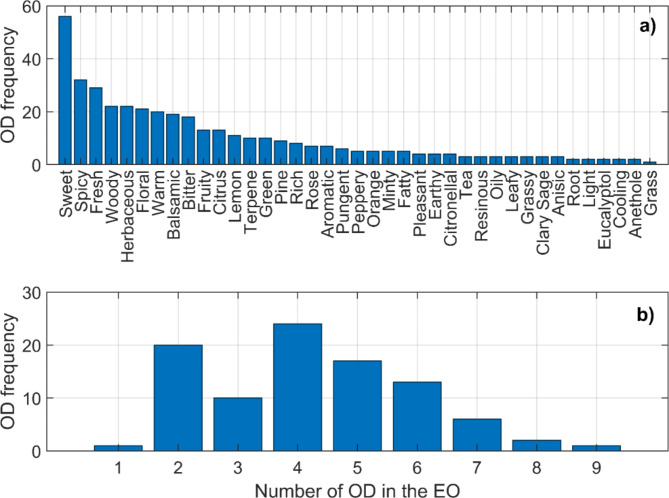
Odor Descriptors in the database. (a) Odor Descriptor frequency in the database (b) Distribution of the number of Odor Descriptors in each Essential Oils.

Odor profiles are highly subjective and prone to context dependency^30,31^ so databases of odors tend to be noisy with an inherent high level of uncertainty. In this study, mass spectrometry data from 50 m/z to 200 m/z were collected from 94 commercially available Essential Oils (EO)^32^. Mass spectrometry data can be obtained from samples without prior knowledge of the odorant chemicals, unlike other methods that rely on chemico-physical parameters to predict odor descriptors^11–13^. Also, odor descriptors representing the scent profiles of the 94 essential oils were compiled (Figure S1). These descriptors, identified by experts, employ specific terminology to classify and describe various odor qualities. The dataset includes 39 distinct odor descriptors, covering a wide range of scents from general categories such as “Sweet” and “Floral” to more specific ones like “Resinous’’ and “Balsamic”. Figure 2.a details the odor descriptors along with their frequency of occurrence within the essential oils dataset, whereas Fig. 2.b illustrates the distribution of the number of odor descriptors associated with each essential oil. Odor descriptor information was encoded into a matrix of 94 rows, one for each essential oil, and 39 columns, each corresponding to a specific odor descriptor (Figure S1). The matrix entries were assigned binary values, with zero indicating the absence and one indicating the presence of a particular odor descriptor in an essential oil (the procedure of mass spectrum measurement is detailed in Methods). In summary we collected data from 94 essential oils, each with mass spectrometry data with 201 points and odor descriptor data consisting of 39 binary values.

## Odorants mixing algorithm

The algorithm described in this paper mixed the essential oils at different ratios to reproduce a desired target odor descriptor set. It has three key elements: a DNN that predicts the odor descriptor set from a given MS, a set of 20 odor components that simplify the essential oil mixing process, and an iterative gradient descent algorithm that aims to minimize the distance between a target desired odor descriptor set and the odor descriptor set predicted by the DNN. The algorithm adjusts the odor component mixture ratios to minimize the distance between the two odor descriptor sets. Each of these key elements is described in the next subsections.

### Deep neural network to predict the odor descriptor set

**Fig. 3:**
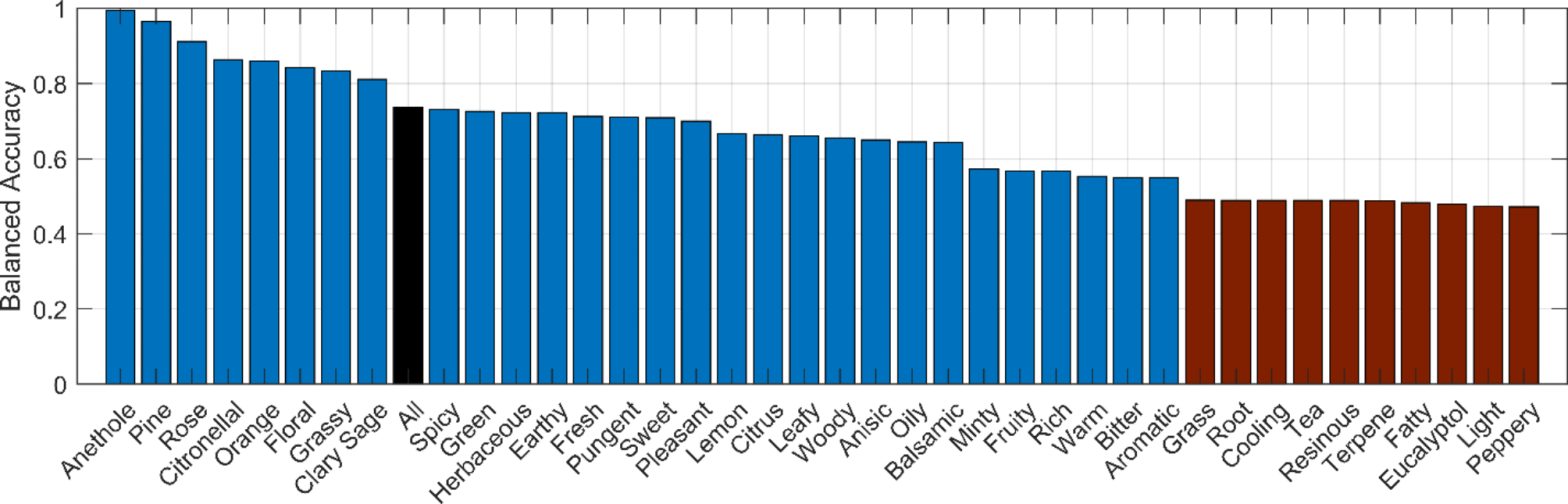
Balance accuracy of every odor descriptor. The blue bars correspond to odor descriptors with balance accuracy above 0.5 and red below 0.5. The “All” odor descriptor is in black.

In the present study, a DNN^21,22^ was utilized to predict qualitative odor descriptors (ODs) set from quantitative mass spectrum data extending the findings from our group’s prior research^19,33^ which demonstrates the capability of DNNs to predict odor ratings based on MS data of individual odorous chemical compounds. In this work, the DNN input was a 201-dimensional vector representing the MS, and the output was a 39-dimensional vector of odor descriptors (Figure S5). The main characteristics of the DNN were 4 hidden layers with strong regularization through dropout and data augmentation to address the limited number of training points and prevent overfitting. Additional details of the network architecture and the training can be seen in Methods. The validity of the DNN training method and its architecture were tested with leave-one-out validation and was evaluated by the balanced accuracy since the odor descriptors frequency were highly imbalanced. Balanced accuracy gives a better assessment than accuracy as the latter tends to overestimates performance by giving too much weight to true negatives. The total balanced accuracy was 0.736, as shown in Fig. 3 where the balanced accuracy of each individual odor descriptor is also displayed. Figure S8 shows the detailed predictions and errors of the DNN of all the cross-validated essential oils.

### Odor components

Using the mass spectra of 180 essential oils, that includes the 94 essential oils with odor descriptor data and 86 without odor descriptor data, 20 odor components were calculated using NMF^10^ so 180 essential oils were used to calculate the odor components while 94 essential oils were used to predict the odor descriptors. The number of odor components was set to 20, aligning with the future goal of implementing this algorithm in an olfactory display device equipped with 20 channels^25^. These odor components acted as basic odors that could be combined to reproduce other odorous compounds. Each odor component consists of a sparse mixture of 180 essential oils. They were calculated so that we can reconstruct the MS of the 180 essential oils with minimal errors. They were calculated by non-negative matrix factorization minimizing the Kullback-Leibler (KL) divergence between the MS of the essential oils and the MS of the reconstruction of the essential oils with the odor components. Once calculated they allow the reproduction of the 94 essential oils MS with a RMSE of 0.0011, indicating that the mass spectra were almost entirely reconstructed using only 20 odor components, as the error was negligibly small. Figure S10 shows the mass spectrum reconstruction errors when a non-negative least squares regression was used to approximate the 94 essential oils mass spectra with the odor components used together with the DNN.

### Algorithm for odor composition

An algorithm was designed to generate a recipe of Essential Oil (EO) mixtures that produces a desired Odor Descriptor (OD) set. The process, detailed below and illustrated in Figure S12, involves several steps. As part of the setup a chosen desired set of odor descriptors is used as input to the algorithm, referred to as target odor descriptor set. In step 1 an initial uniform combination of the Odor Components (OCs) is used as the starting recipe, so the 20 odor components are mixed at the same rates. Then the iterative process begins; in the step 2 the MS of that current odor component mixture is calculated by linearly mixing the mass spectra of the odor components in the same proportion, since the MS was considered a linear superposition. In the next step 3 the DNN is used to predict the odor descriptor set for the current component mixture using the calculated MS as input. In step 4, the distance between the predicted odor descriptor set and the target odor descriptor set is calculated using Mean Squared Error (MSE). In step 5 the distance between the odor descriptor sets is used as a loss for a gradient descent search. The gradient of the RMSE with respect to the odor component recipe mixing ratios is calculated, propagating through the DNN up to the odor component ratio mixing by the derivative chain rule (see methods and Figure S13). In each iteration the odor component mixing ratio is modified to minimize the distance between the two odor sets. The procedure is very similar to training a neural network, but in this case only the mixing ratios of the odor components are updated.

**Fig. 4:**
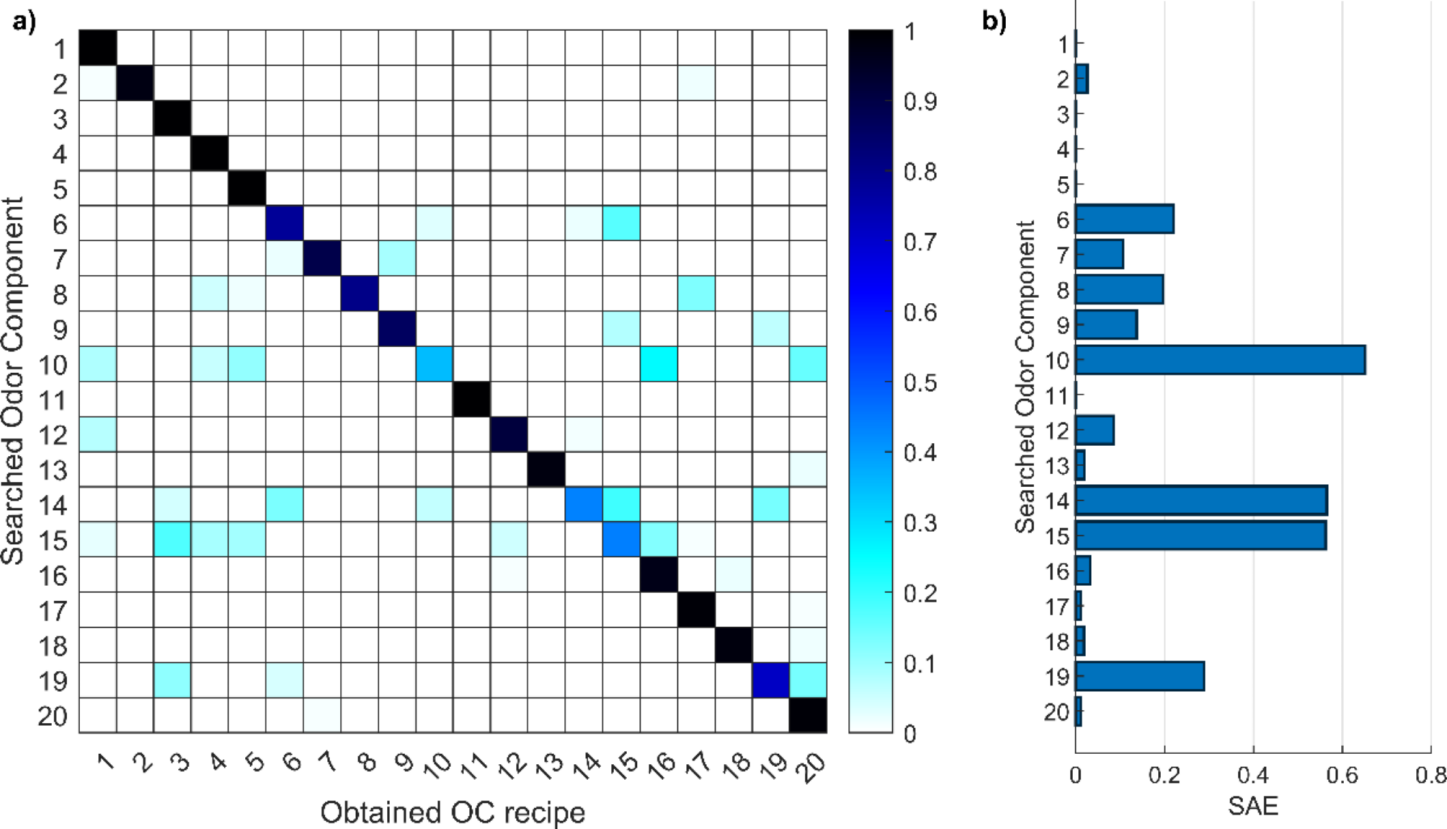
Errors on the search algorithm when searching the odor descriptors of the odor components. (**a**) The odor component mixture obtained (**b**) errors on the odor descriptors obtained.

To test whether the algorithm was able to converge correctly, the odor descriptor set of each odor component (as predicted by the DNN) was used as the target odor descriptor set (Figure S11). The result of the 20 searches can be in Fig. 4.b where the Sum of Absolute Errors (SAE) of the odor descriptor set is depicted. The Figure shows that the algorithm effectively converges to the odor descriptor sets with minimal errors across the majority of the odor components. For five odor components, the observed error exceeded 0.2, and it did surpass 0.6 in one instance (for odor component 10). Figure 4.a shows the odor component recipes obtained. The higher errors of the odor component mixture ratios compared to the lower error rates associated with odor descriptor set reconstruction suggest that, despite the algorithm’s precise convergence to the target odor description sets, it may occasionally converge to a local minimum. Nevertheless, the low error observed in the odor description obtained indicates that the algorithm remains effective for converging to odor descriptor sets, even in when it converges to a local minimum. The algorithm performed well in reconstructing most odor profiles with low errors, but without ground truths for the generated recipes, the computational assessments were only indirect. Sensory testing is crucial for a definitive validation, as human perception is needed to confirm whether the created odors matched the intended odors. So, to validate the numerical results, two sensory tests were conducted.

## Sensory test

Four different essential oils were chosen as initial points. Their odor descriptors were used to generate four replicas with the gradient descent search, named “Replicated”. Then for each essential oil, one odor descriptor among “Floral”, “Sweet”, “Fresh” and “Woody” was added to their odor descriptors set and a new gradient descent search was done. The two first columns of Table 1 show the different combinations of essential oil and odor descriptor added. The essential oils and the odor descriptor added were chosen in base of the DNN balanced accuracy prediction from the leave-one-out cross validation. Also, the selection of modification was limited to searches of recipes that achieved a high level of convergence between the target odor descriptor set and the obtained odor descriptor set.

In the first sensory test, panelists were presented with two samples: a “Replicated” essential oil and a modified version with an added specific odor descriptor (e.g., “Floral”). The goal for each panelist was to correctly identify which of the two samples had the added odor descriptor. The test was conducted using a two-alternative forced-choice (2AFC) method^20^, where panelists were required to choose between two options, ensuring no neutral responses were possible. The evaluation process was double-blind, meaning neither the panelists nor the administrators knew which sample was the original or the modified version. This eliminated potential biases and ensured objective results. The two samples were presented in randomized order to avoid any sequence effects influencing the panelists’ decisions.

The test was repeated eight times for each panelist, with the order of presentation randomized in each trial. A chi-square test was applied to determine whether the panelists’ ability to identify the modified sample significantly deviated from random chance^20^. The results of each trial were aggregated for each specific added odor descriptor (e.g., Floral) and overall, providing both descriptor-specific and general outcomes (as shown in Table 1).

**Table 1:** Sensory test 1 results.

EO	Added OD	Correct	Incorrect	Correct	Incorrect	Chi-square	*p*
Chamomile	Floral	10	5	21	9	4.80	0.029
Citrus A.B.		11	4				
Cedarwood	Sweet	12	7	25	13	3.79	0.052
Origanum		13	6				
Cedarwood	Fresh	11	8	22	16	0.95	0.330
Origanum		11	8				
Chamomile	Woody	7	8	13	17	(0.43)	(0.465)
Citrus A.B.		6	9				
Aggregated				**81**	**55**	**4.971**	**0.026**

Final aggregated values are in [bold].

In the second sensory test, the same set of samples was used: the “Replicated” essential oil and the modified version with an added odor descriptor (e.g., “Floral”). However, unlike the first test, a duo-trio test was employed^20^. In this setup, panelists were first provided with the original essential oil as a reference sample. Their task was to smell both the “Replicated” and the modified sample, then determine which of the two was perceptually closer to the reference essential oil. This test aimed to assess whether the generated recipe for the “Replicated” sample, reconstructed using calculated odor components, was a better match to the real essential oil than the modified sample. The duo-trio test offers a comparative method, requiring panelists to identify subtle differences or similarities between the samples and the reference. As with the first test, this sensory evaluation was conducted in a double-blind manner, with random presentation of the two samples (“Replicated” and modified). A chi-square test was again used for statistical analysis^20^. The responses from the panelists were aggregated overall providing a general outcome (as shown in Table 2). This allowed us to determine whether the generated “Replicated” recipe was consistently chosen as closer to the real essential oil compared to the modified sample, providing evidence of the accuracy of the reconstruction process.

**Table 2:** Sensory test 2 results.

EO	Added OD	Correct	Incorrect	Chi-square	*p*
Chamomile	Floral	12	3	5.40	0.020
Citrus A.B.		13	2	8.07	0.0045
Cedarwood	Sweet	12	3	5.40	0.020
Origanum		11	4	3.27	0.071
Cedarwood	Fresh	9	6	0.60	0.44
Origanum		13	2	8.07	0.0045
Chamomile	Woody	13	2	8.07	0.0045
Citrus A.B.		9	6	0.60	0.44
Aggregated		**92**	**28**	**34.13**	**< 0.001**

Final aggregated values are in [bold].

## Discussion

In this study the DNN, trained with data of 94 essential oils, had an overall balanced accuracy of 0.736 in predicting odor descriptors (Fig. 3), consistent with other studies on odor descriptor predictions^15,35,36^. The balanced accuracy prediction was independent of the frequency of the odor descriptors in the database but there was a small positive correlation with the number of odor descriptors in each essential oil. This is because the more odor descriptors an essential oil had, the more information was available for prediction (Figure S14). The balanced accuracy varied across different odor descriptors, high for some of them such as “Floral” whose balanced accuracy was 0.843, or lower for “Fresh”, “Sweet”, and “Woody” whose balanced accuracy were 0.712, 0.709, and 0.655 respectively.

Regarding the sensory test, although it evaluates only a limited number of odor descriptors, creating a completely new odor and determining its odor descriptors by untrained panelists in a “check all that apply” test is a very challenging task. Such an approach would introduce uncertainty on the panelist’s assessment. On the other hand, to make a replication of one of the essential oils used in the DNN’s training (based on its odor descriptors), presenting a mass spectrum that is essentially the reconstruction of that mass spectrum with the odor components is a trivial solution. Furthermore, the reproduction of essential oils using odor components has already been successfully demonstrated in previous studies^10,25,37^ and is not the primary focus of this paper. Instead, this study presents an aroma creation method that adds one odor descriptor and two sensory tests that have a balance between the simplicity of a reliable sensory evaluation and the meaningful creation of new aromas.

The analysis of the first sensory test results for different odor descriptors demonstrated different levels of statistical significance. Specifically, the outcomes associated with “Floral” were statistically significant at the 5% significance level. However while the numbers of correct classifications were higher for “Sweet” and “Fresh” they did not reach the standard significance threshold. “Woody” on the other hand, produced less favorable results, with more incorrect than correct classifications. These variations in outcomes are correlated with the balanced accuracy of the DNN for each specific odor descriptor, as illustrated in Fig. 3. An explanation of this is that since the DNN holds the relationships of the mass spectrum data and the odor descriptors, a high accuracy was necessary to the successful creation of the new recipes. Improving the accuracy of each odor descriptor, by adding additional data or by improving the algorithm design, would greatly benefit the overall performance of the algorithm presented in this study.

Moreover, aggregating all the experiments the procedure shows a significance of *p* = 0.0258. This increase of significance compared to the significance of the individual odor descriptors was caused by the increased number of experiments that increased the strength of the statistical test. These results denote that the algorithm can in fact add odor descriptors to a predetermined odor descriptor set.

The second sensory test provided additional evidence supporting the accuracy of the generated odors in replicating the essential oils. Panelists consistently identified the “Replicated” samples as being perceptually closer to the reference essential oil compared to those with added odor descriptors. This suggests that the generated recipes successfully captured the essential oil’s original olfactory profile more accurately than the modified versions, which introduced additional odor notes.

Future studies could explore several areas to enhance the algorithm. First, a more optimized network could be developed, as the network used in this study was not fully optimized, and its hyperparameters were selected heuristically. Improvements would also benefit from additional data, as the current dataset is limited to only 94 essential oils. Furthermore, the network was validated using a leave-one-out approach, which is known to overestimate performance. However, due to the small number of essential oils and the high dimensionality of the input and output, we opted to retain as many samples as possible. Removing any essential oils would further reduce the already limited data available for predictions.

As mentioned in the paper, the implementation of this algorithm enables practical use with a 20-channel odor display^25^. Although the gradient search takes a couple of minutes on a standard computer, it is suitable for dynamic displays, such as those used alongside other media or in recreational environments. The proof of concept presented can be implemented and tested with a limited number of odor components. Additionally, techniques that collect user feedback regarding odor satisfaction can serve as additional training data, enhancing the DNN’s accuracy through retraining, fine-tuning, or reinforcement learning. In conclusion our cheminformatics approach can create scent with intended odor profiles although the accuracy should be improved. The designed algorithm uses a DNN to predict odor descriptors from mass spectrum data, then a gradient descent search uses this DNN to find new odorous recipes. Additionally, the use of odor components, derived from non-negative matrix factorization, simplify the algorithm by limiting the ingredients of the recipes to 20 enhancing the algorithm’s practicality. The validity of the scent creation process was confirmed through sensory evaluations conducted with non-expert panelists, affirming the algorithm’s capacity to generate novel odors.

This research contributes to the advancement of the scent digitalization technology by demonstrating the potential of Deep Neural Networks in the domain of odor design, it enriches the research on olfactory digitization and also opens the field for further innovation in digital scent synthesis.

Methods.

## Essential oils data

Essential oils were bought from Absolute Aromas, Zefir, Pranarom International, Palm Tree, and Naturas Psychos. Their odor descriptors were compiled and curated manually from^32^ and Wikipedia. The essential oils 50 to 250 m/z mass spectrometry data were measured by a Gas Chromatography/Mass Spectrum (GC/MS), Agilent Technologies B5977 MSD coupled with 7890B GC. The carrier gas was 99.99995% helium, the temperatures at the injector, column, and auxiliary port were set to 250 °C but the measurement procedure bypassed the GC. The MS had 70 eV electric ionization and was set at 230 °C. Each sample was diluted with ethanol (99.5% purity) to 9:1 v/v. Each sample was measured for 6 to 7 min 10 times. The 10 measurements were averaged and pretreatment was employed to remove noise^37^. Figures S2, S3, and S4 in the supplementary materials disclose the correlation between essential oils’ mass spectra, its effects on odor components, and its effects on the DNN’s prediction of odor descriptors.

### DNN

The parameters of the DNN were: Input: size of 201. Layer 2: fully connected (size of 4754) with leaky ReLU (0.01 slope). Layer 3: fully connected (size 3565) with leaky ReLU (0.01 slope). Layer 3: fully connected (size 2377) with leaky ReLU (0.01 slope) and dropout of 0.9. Layer 4: fully connected (size 423) with leaky ReLU (0.01 slope) and dropout of 0.75. Layer 5: Output layer (size of 39) with leaky ReLU (0.01 slope). The loss function was MSE with L1 regularization with coefficient of 0.005. Figure S5 shows a scheme of the network. The training parameters were algorithm: ADAM, initial Learning Rate: 0.001, Learn Rate Drop Factor: 0.995 each epoch, Gradient Decay Factor: 0.9, Squared Gradient Decay Factor: 0.999, Minibatch size: 150, Layer L2 regularization: 1e-4, Max. Epochs: 1000. Since most of the odor descriptors of combined chemical compounds follow a superposition^38^, data augmentation consisting of adding mixtures of two essential oils (linearly mixing both essential oils in mass spectrum and in the odor descriptors at random ratios) was used. Also, adding a 10% uniform noise to each mass spectrum was utilized to improve generalization. The augmented samples were generated randomly at each training step, this together with the dropout helped to overcome overfitting as detailed in supplementary materials in Neural network and training Figures S6, S7, S8, and S9.

## Odor components

The 20 odor components (Table S1 presents the first three odor components; the remaining components are available in the data repository) were determined using Non-negative Matrix Factorization (NMF) as expressed by Eq. (1), where *MS* is the Mass Spectra of the Essential Oils (the dimensions corresponding to 180 Essential oils and 201 lines of the Mass Spectra), *W* the matrix of the ratios of the Essential Oils in each odor component (20 odor components and 180 essential oil), and *H* are the MS representation of such odor components (20 odor components and 201 MS lines). The function described in Eq. (2) was used as the loss function (*L*_*KL*_). It was based on the Kullback-Leibler (KL)^39^ divergence between the reconstructed MS of 180 essential oils and their original MS data^10^.

1 $$\:MS_{{\left( {180 \times 201} \right)}} = W_{{180 \times 20}} \times H_{{20 \times 201,}}$$

2 $$\:{L}_{KL}={\sum\:}_{i=1}^{180}{\sum\:}_{j=1}^{201}({MS}_{i,j}{log\left(WH\right)}_{i,j}-{log\left(WH\right)}_{i,j})$$

## Gradient descent search

The gradient descent search was done using the MATLAB deep learning toolbox by defining new custom layers and employing the Stochastic Gradient Descent algorithm. The detailed scheme of the calculations can be seen in Figure S13. The forward pass used to predict the odor descriptor set of an odor component mixture is detailed from left to right: First there is the ratio of odor components, that is the recipe we are trying to find. Only this 20-dimension vector will be modified during the gradient descent search. The second step is a normalization so the sum of the elements is 1, denoted by *OC*^*n*^. We multiply those ratios with the matrix *H*, from Eq. (1), to obtain the overall *MS* of the odor component mixture. This *MS* is normalized so its maximum value is 1, denoted by *MS*^*n*^. Then *MS*^*n*^ is used to predict the odor descriptors with the trained DNN. The goodness of the predictions was evaluated by the loss function given by Eq. (3) were *MSE* means Mean Squared Error, $\:\widehat{\overrightarrow{OD}}$ is the predicted Odor Descriptors, $\:{\overrightarrow{OD}}^{t}$ is the target, real odor descriptors, and *i* is the index denoting each element of the odor descriptor set.

3 $$\:L\left(\widehat{\overrightarrow{OD}}\right)=\:MSE\left({\overrightarrow{OD}}^{t},\:\widehat{\overrightarrow{OD}}\right)=\:\frac{1}{n}\sum\:_{p}{({OD}_{p}^{t}-{\widehat{OD}}_{p})}^{2}$$

To calculate the gradient of the loss function with respect to the *OC* the derivative chain rule was used as described by Eq. (4), where *p*,* q*,*r*,* s*,*i* represent the different elements of the vectors denoted.

4 $$\:\frac{\partial\:L}{\partial\:{OC}_{i}}=\sum\:_{p,q,r,s,i}\frac{\partial\:L}{\partial\:{\widehat{OD}}_{p}}\frac{\partial\:{\widehat{OD}}_{p}}{\partial\:{MS}_{q}^{n}}\frac{\partial\:{MS}_{q}^{n}}{\partial\:{MS}_{r}}\frac{\partial\:{MS}_{r}}{\partial\:{OC}_{s}^{n}}\frac{\partial\:{OC}_{s}^{n}}{\partial\:{OC}_{i}}$$

Each of the different elements of Eq. (4) are detailed below in Eq. (5) to (9), where in Eq. (7) *m* represent the index of the element that is maximum in the *MS* vector.

5 $$\:\frac{\partial\:L}{\partial\:{\widehat{OD}}_{p}}=\:2({OD}_{p}^{t}-{\widehat{OD}}_{p})$$

6 $$\:\frac{\partial\:{\widehat{OD}}_{p}}{\partial\:{MS}_{q}^{n}}=\frac{\partial\:DNN}{\partial\:{MS}_{q}^{n}}$$

7 $$\:\frac{{\partial \:MS_{q}^{n} }}{{\partial \:MS_{r} }} = \frac{{\partial \:\left[ {\frac{{MS_{q} }}{{MS_{m} }}} \right]}}{{\partial \:MS_{r} }} = \left\{ {\begin{array}{*{20}l} {for\:q = r = m:\:} \hfill & {\frac{{\partial \:}}{{\partial \:MS_{r} }}\left[ {\frac{{MS_{r} }}{{MS_{r} }}} \right] = 0} \hfill \\ {for\:q \ne \:r;q = m:} \hfill & {\frac{{\partial \:}}{{\partial \:MS_{r} }}\left[ {\frac{{MS_{m} }}{{MS_{m} }}} \right] = 0} \hfill \\ {for\:q \ne \:r \ne \:m:} \hfill & {\frac{{\partial \:}}{{\partial \:MS_{r} }}\left[ {\frac{{MS_{q} }}{{MS_{m} }}} \right] = 0} \hfill \\ {for\:q = r \ne \:m:} \hfill & {\frac{{\partial \:}}{{\partial \:MS_{r} }}\left[ {\frac{{MS_{r} }}{{MS_{m} }}} \right] = \frac{1}{{MS_{m} }}} \hfill \\ {for\:q \ne \:r;r = m:} \hfill & {\frac{{\partial \:}}{{\partial \:MS_{m} }}\left[ {\frac{{MS_{q} }}{{MS_{m} }}} \right] = \frac{{ - MS_{q} }}{{MS_{m} ^{2} }}} \hfill \\ \end{array} } \right.$$

8 $$\:\frac{\partial\:{MS}_{i}}{\partial\:{OC}_{j}^{n}}={H}_{i,j}$$

9 $$\:\frac{\partial\:{OC}_{s}^{n}}{\partial\:{OC}_{i}}=\frac{\partial\:\left[\frac{{OC}_{s}}{{\sum\:}_{k}{OC}_{k}}\right]}{\partial\:{OC}_{i}}=\:\left\{\begin{array}{l}s=i:\:\frac{\partial\:}{\partial\:{OC}_{s}}\left[\frac{{OC}_{s}}{{R}_{s}+{\sum\:}_{k\ne\:s}{OC}_{k}}\right]=\frac{1}{{\sum\:}_{k}{OC}_{k}}+\frac{-{OC}_{s}}{{\left({\sum\:}_{k}{OC}_{k}\right)}^{2}}\\\:s\ne\:i:\frac{\partial\:}{\partial\:{OC}_{i}}\left[\frac{{OC}_{s}}{{OC}_{i}+{\sum\:}_{k\ne\:i}{OC}_{k}}\right]=\frac{-{OC}_{s}}{{\left({\sum\:}_{k}{OC}_{k}\right)}^{2}}\end{array}\right.$$

### Sensory test

For the preparation of the samples for the sensory test, first the odor components were prepared by pipetting essential oil into 5 µl vials. Then the odor components were mixed in the ratios obtained from the algorithm into 35 ml vials. To mitigate possible odor saturation, the essential oils were diluted in ethanol 1:10, reducing their odor intensities. 26 untrained subjects were recruited from the Tokyo Institute of Technology (21 to 64 years old). The sensory test was done under the approval by the human subject research ethics review committee of Tokyo Institute of Technology in accordance with Helsinki declaration (Approval Number 2023027). Informed consent was obtained from all participants.

## Electronic supplementary material

Below is the link to the electronic supplementary material.

Supplementary Material 1

## Data Availability

The associated data, including inputs and the output odor component mixes are available at OSF repository: https://osf.io/frm5z/?view_only=05f85daaa78c43a7847dfde280f65a58. Additional data and code can be made available upon request to Nakamoto Takamichi (nakamoto.t.ab@m.titech.ac.jp).
