# Supplementary material for: Automatic scent creation by cheminformatics method
Source: Sci Rep. 2024 Dec 28;14:31284. doi: 10.1038/s41598-024-82654-7 (PMC11682350; doi:10.1038/s41598-024-82654-7)
Supplement: Supplementary file 1 — Supplementary Material 1 [file 41598_2024_82654_MOESM1_ESM.docx]

Supplementary materials

### Detailed sensor data Sensory


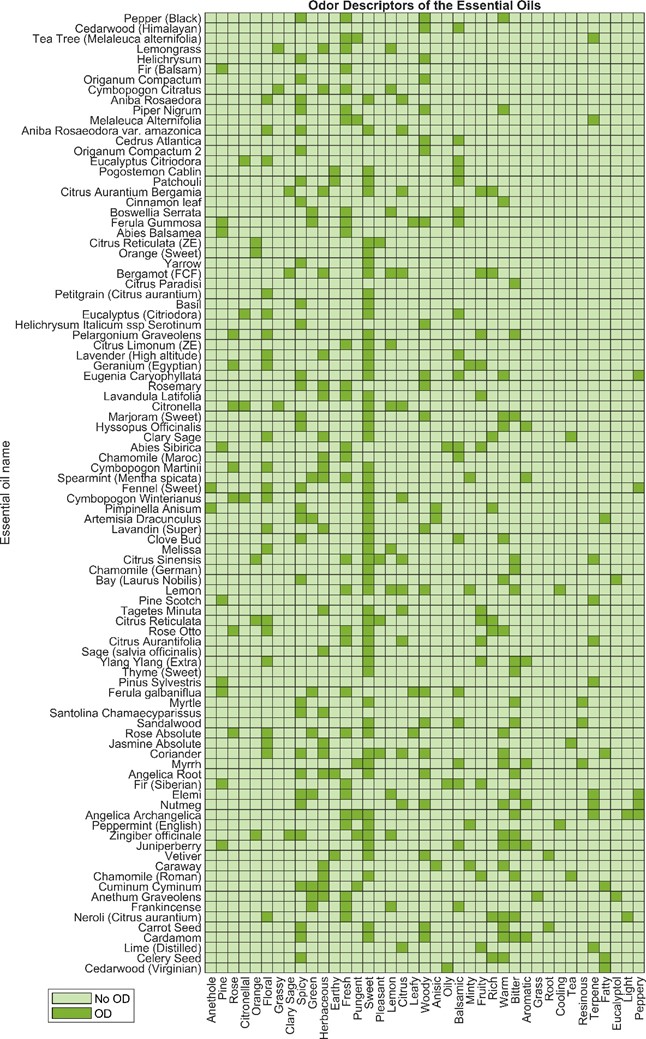


Figure S1. Essential oils and their odor descriptors used in the training of the Deep neural network.

### Correlation among the essential oils.

We calculated the correlations of the mass spectra for the essential oils, obtaining a mean correlation of 0.5757 between each pair of oils. Figure S2a displays the correlation matrix, while Figure S2b shows the histogram of these correlations. The strongest correlations are typically between oils from different manufacturers or oils that belong to the same botanical family (such as lavender and clary sage, or different varieties of orange). One constraint in our Non-negative Matrix Factorization (NMF) analysis was to limit the number of basis vectors to 20. While it might seem ideal to select odor components specific to each essential oil cluster for reconstruction, this approach may not be optimal with a restricted component count. This is evident in Figure S3, where the PCA plot shows the distribution of odor components around essential oils without forming distinct clusters of similar oils.


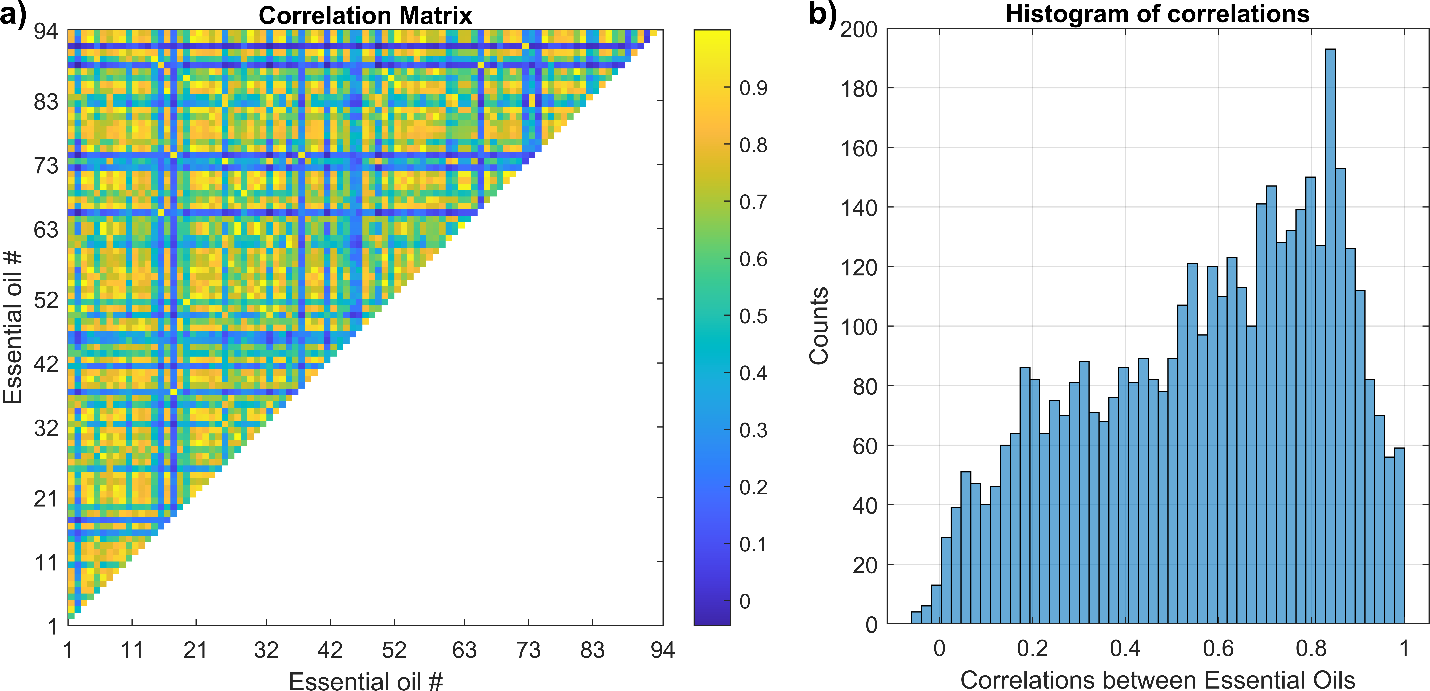


Figure S2. a) Correlation matrix for essential oils based on the mass spectrum data. b) Histogram of the correlations.


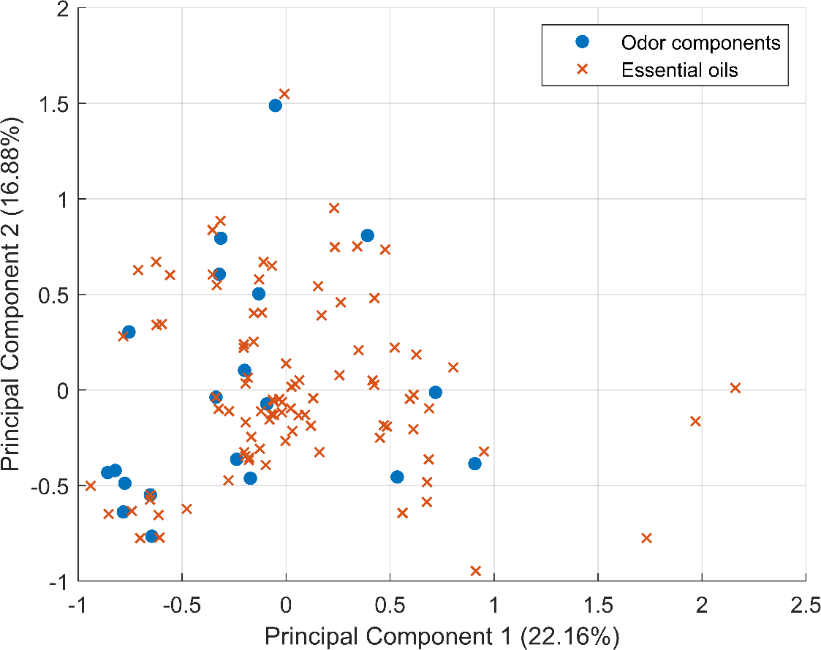


Figure S3. PCA of Mass Spectra for Odor Components and Essential Oils

Given the high correlations observed between some essential oils, we further investigated their impact on the deep neural network (DNN) predictions using a leave-one-out cross-validation approach. Figure S3 presents the balanced accuracy of the odor descriptors prediction of the DNN of the 94 essential oils plotted against the maximum correlation of the essential oils. If the network relied on the most similar essential oil for prediction, we would expect higher balanced accuracy for higher correlations. However, the correlation of 0.3395 (as seen in Figure S4) indicates a weak relationship between essential oil similarity and DNN prediction accuracy. Moreover, when essential oils with correlations higher than 0.9 were removed, the overall correlation dropped from 0.3395 to 0.2687, further weakening the relationship between highly correlated essential oils and DNN performance.

Figure S4. Scatter plot and linear fit of the Balanced accuracy versus the maximum correlation for each essential oil.

### Neural network and training

Figure S5 shows the structure of the network. The structure and hyper-parameter selection of the DNN were optimized heuristically based on our experience working with the prediction of odors and odor ratings from mass spectrum data and it didn’t undergo a systematic optimization process.

Because of the small dataset, steps were taken to mitigate overfitting. The dataset was augmented by adding noise and combining random pairs of mass spectra, which helped prevent overfitting. Strong dropout was applied to the latter layers to ensure robustness during classification and to avoid learning prior cross correlations between the odor descriptors. Both leave-one-out and 10-fold cross-validation methods were applied to assess the network’s performance, given the dataset's small size and high dimensionality (201 inputs and 39 outputs with 94 data points).


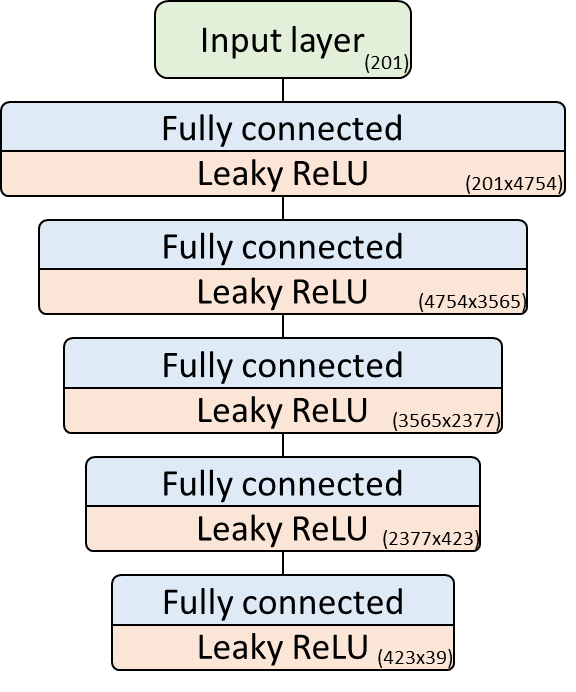


Figure S5. Scheme of the DNN used. In each layer the input and output sizes are denoted by the number in the right bottom such as (Input Size x Output Size)

Figure S6 presents the Root Mean Squared Error (RMSE) for both leave-one-out and 10-fold cross-validation methods. It compares the RMSE values for the augmented training data with those from the validation set of the final fold in the network’s training. The RMSE for the training set is higher due to data augmentation and added noise, whereas the RMSE for the validation set, which does not include the added noise, remains stable throughout training. This stability suggests that overfitting is effectively controlled. Noise injection in the input layer and high dropout rates in the latter layers function as regularization techniques that help prevent the network from overfitting to the augmented data.


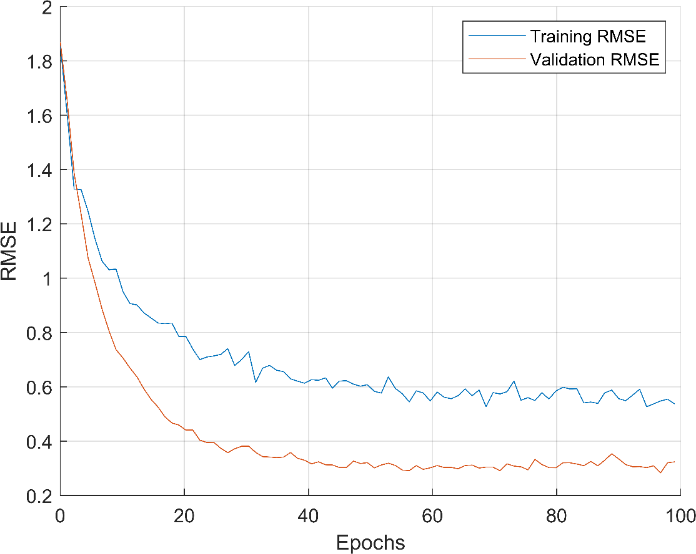

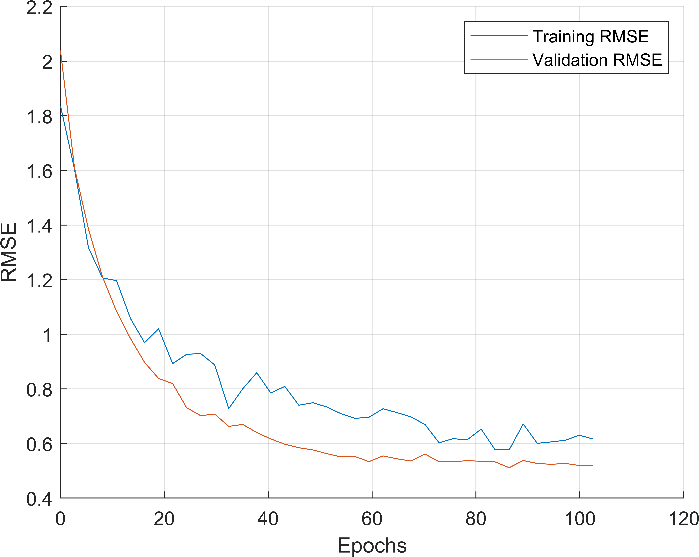


Figure S6. Training and validation Loss and RMSE for one of the leave-one-out validation steps (left) and for one of the 10-fold validation steps (right).

To confirm the robustness of the approach, the ROC-AUC curve was drawn, resulting in an AUC of 0.743 for the leave-one-out and 0.765 for the 10-fold cross validation (Figure S7).


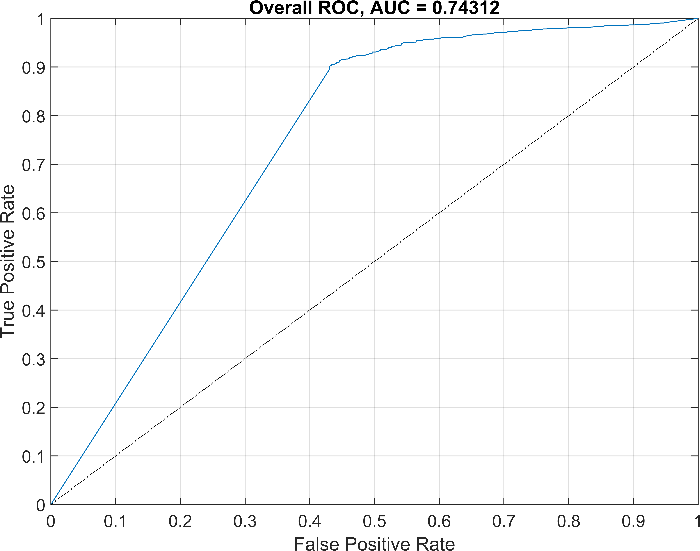


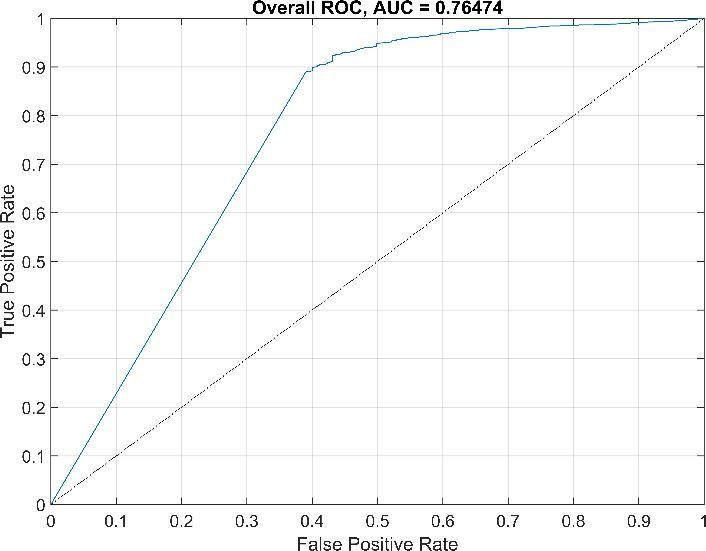


Figure S7. ROC-AUC plot of the leave-one-out validation (up) and for the 10-fold validation (bottom).


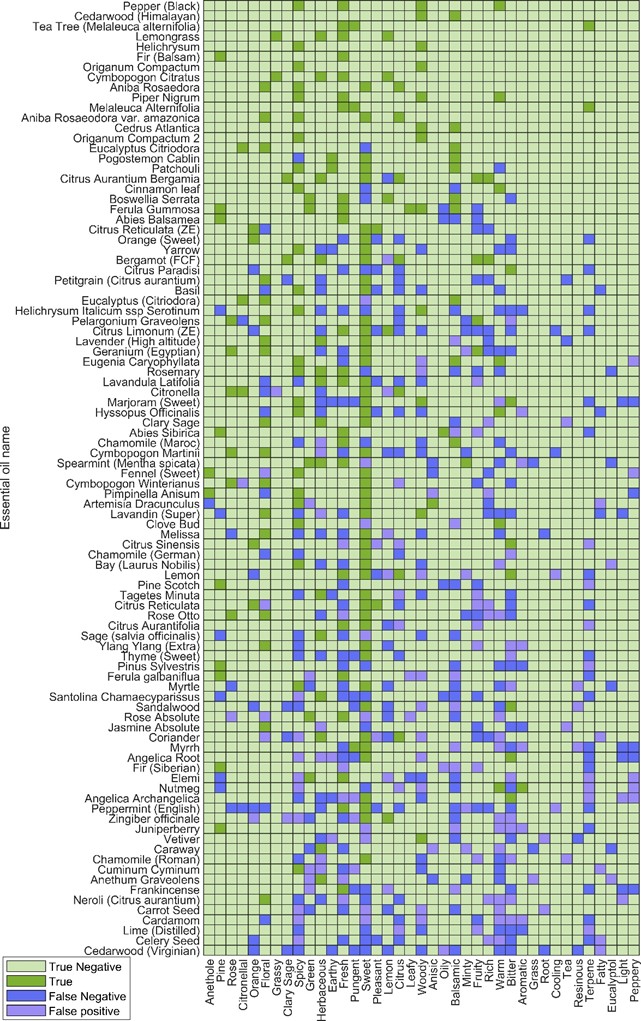


Figure S8. Detailed results of the DNN classification leave one out of validation. The essential oils are reordered from high balanced accuracy (top) to lower balanced accuracy (bottom). The odor descriptors are also re-ordered by balanced accuracy from highest (left) to lowest (right)

In deep neural networks, it is common for networks to have more neurons than inputs. To avoid difficulties with reversing gradient descent when searching for recipes, we limited the number of layers rather than adding depth. In deep networks with many neurons, sparse representations often emerge in the early layers. Figure S9 shows the layer activations, where the layers closest to the input exhibit high sparsity, while weights become more uniformly distributed in layers closer to the output. This sparsity is further promoted by using Leaky ReLU, which allows some negative values to pass through, encouraging sparse activations without completely deactivating neurons.

Figure S9. Histograms of the weighs of the neurons of the different layers of the network.

### Odor components


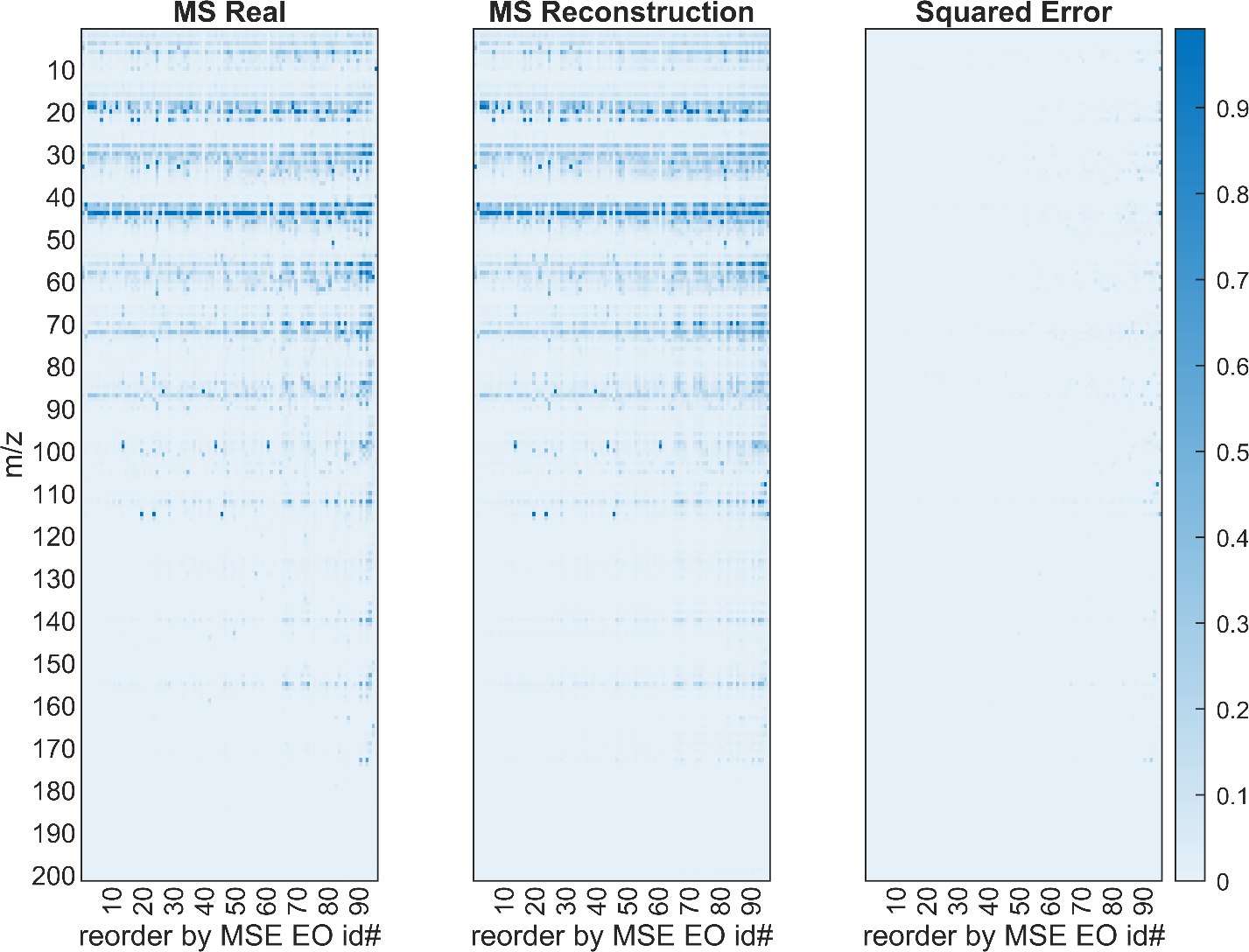


Figure S10. Left) MS of the essential oils, middle) MS of the reconstructed essential oil with the odor component, and right) error of the reconstructions.


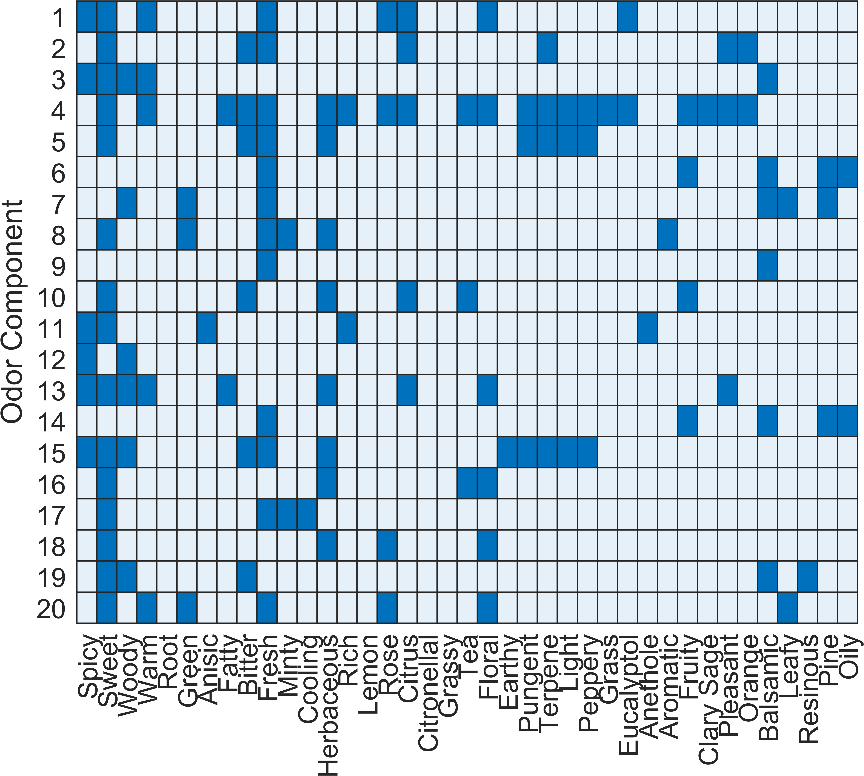


Figure S11. Prediction of the odor descriptor of the Odor components.

### Recipe search algorithm

**
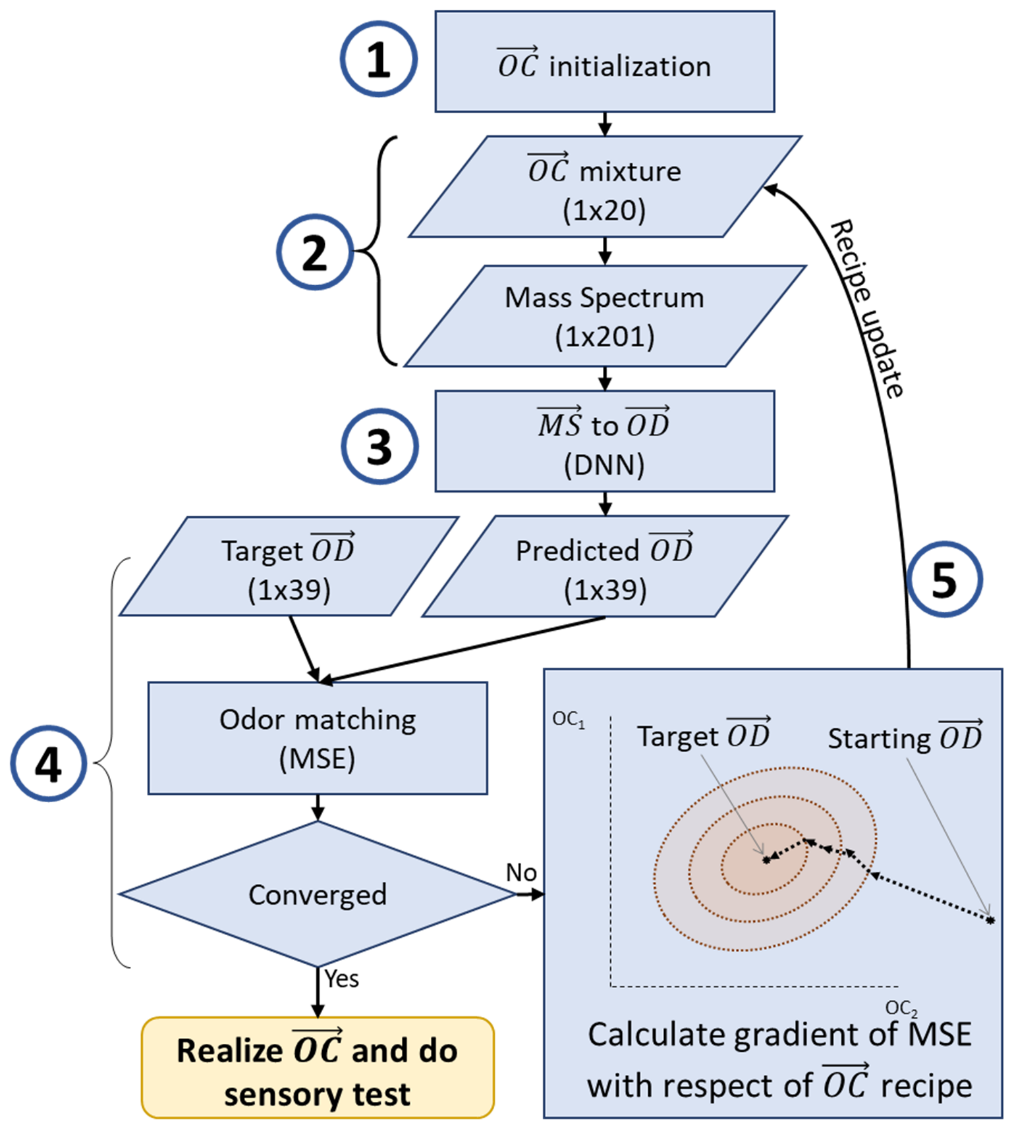
**

Figure S12. Scheme of the gradient descent search algorithm for odor component recipe search. $\vec{OC}$ is the vector that describe the mix of odor components. $\vec{OD}$ is the vector that describe the odor descriptors. $\vec{MS}$ is the vector of the Mass Spectrum data.


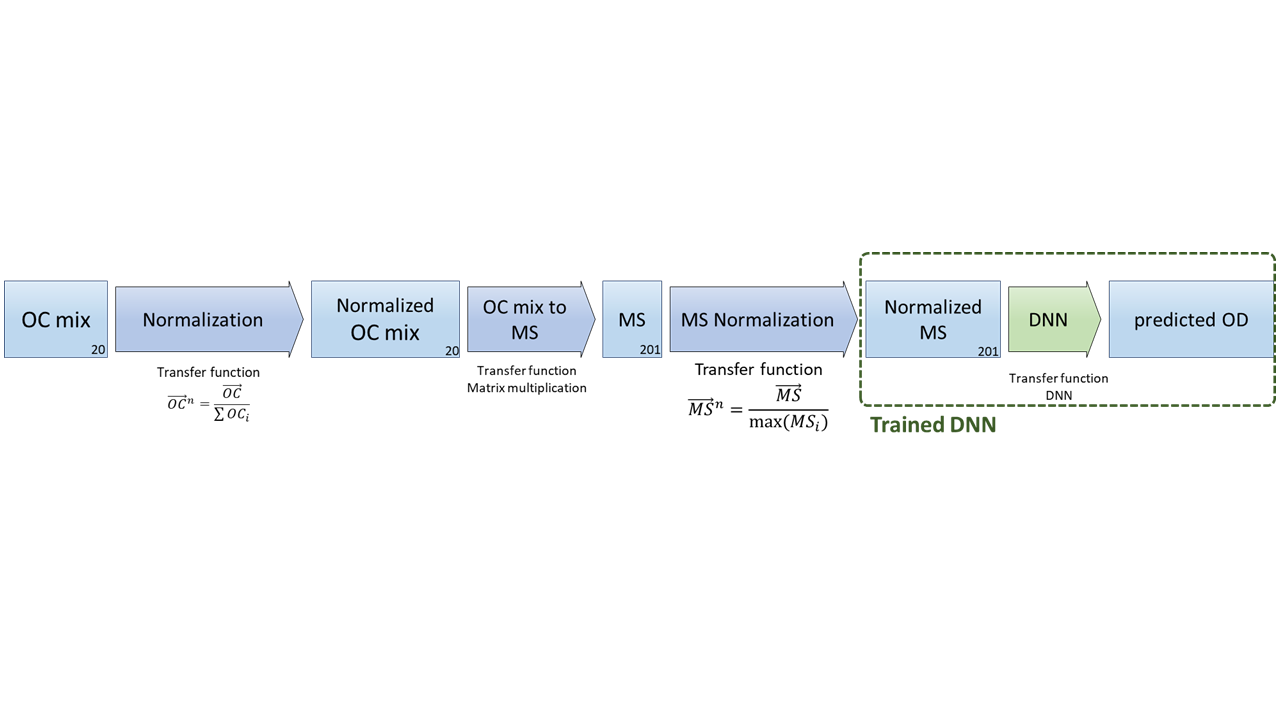


Figure S13. Details of the gradient descent search calculations as detailed in Methods.


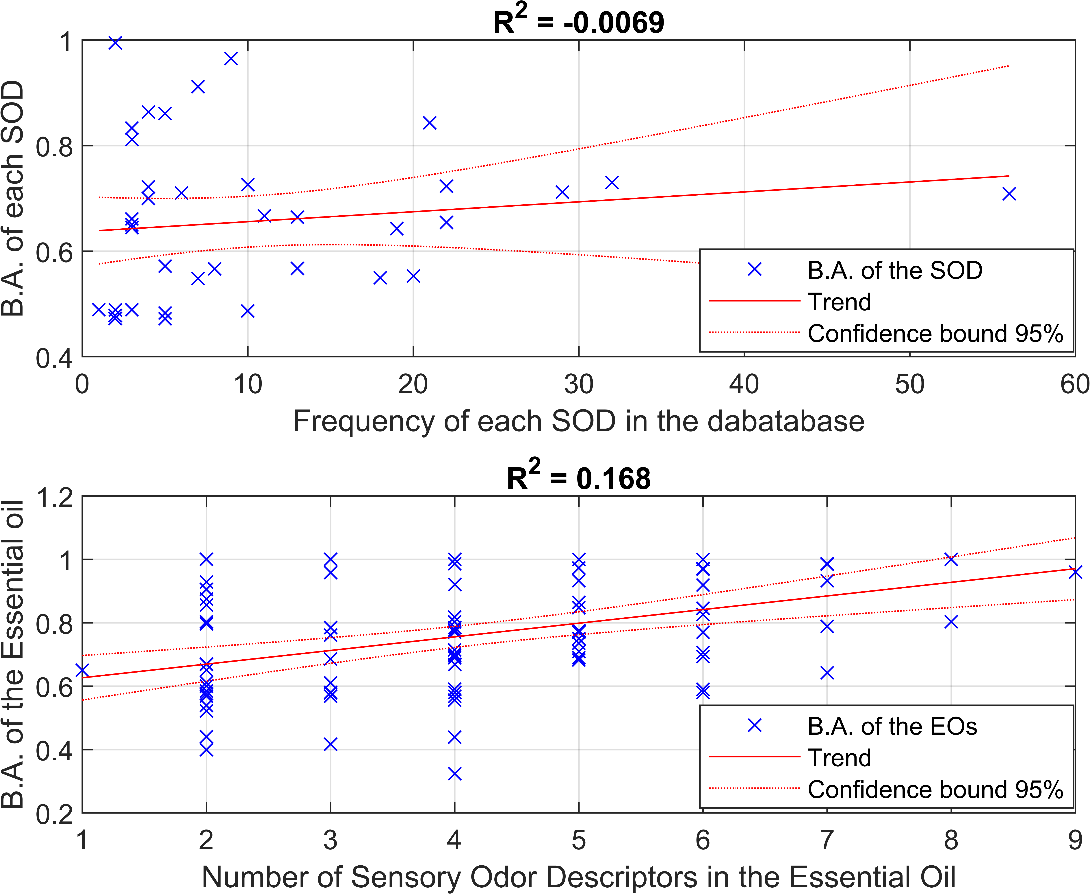


Figure S14. Dependance of the odor descriptor frequency with the balanced accuracy of the DNN. Although there is a small positive correlation between the number of odor descriptors in the database and the balanced accuracy of the odor descriptor, the low R indicates that it is not significant (top). The same conclusion can be drawn from the number of odor descriptors in each essential oil (bottom).

Table S1. Composition of the first three odor component recipes, with each row representing an ingredient (one essential oil) and each column representing an odor component recipe, scaled to a maximum of 100% for each recipe.

| Ingredients of odor component | Odor component | | |
| --- | --- | --- | --- |
|  | 1 | 2 | 3 |
| Peppermint (English) | 0.556 | 0 | 0 |
| Clove Bud | 0 | 0 | 62.6 |
| Citrus Reticulata | 0 | 5.14 | 0 |
| Breatheasy | 31.3 | 0 | 0 |
| Organic Orange | 0 | 94.5 | 0 |
| Melaleuca Minor | 68.2 | 0 | 0 |
| Eugenia Caryophyllata | 0 | 0 | 36.6 |
| Artemisia Dracunculus | 0 | 0.077 | 0 |
| Origanum Compactum | 0 | 0.263 | 0 |
| Oakmoss Absolute | 0 | 0 | 0.791 |
